# Mental health and resilience after the covid-19 pandemic: a multi-ethnic longitudinal survey

**DOI:** 10.1186/s12889-023-17230-1

**Published:** 2023-11-25

**Authors:** Jennifer Sumner, Mark Chen, Alexius Matthias Soh Sheng En, Vanessa Lim Wei Xun, Sin Hui Neo, Yee Wei Lim

**Affiliations:** 1 Department of Medicine, Yong Loo Lin School of Medicine, National University of Singapore, Singapore, Singapore; 2 Medical Affairs – Research Innovation & Enterprise, Alexandra Hospital, National University Health System, 378 Alexandra Road, Singapore, 159964 Singapore; 3 National Centre of Infectious Diseases, Singapore, Singapore

**Keywords:** Mental health, Public health, COVID-19, Pandemics

## Abstract

**Background:**

Longitudinal work on the impact of COVID-19 on population mental health and resilience beyond the first year of the pandemic is lacking. We aimed to understand how mental health and resilience evolved during the pandemic (2020) and two years later (2022) in a multi-ethnic Singaporean population. In addition, we assessed what characteristics were associated with mental health and resilience scores.

**Methods:**

We surveyed and analysed two balanced panel samples up to four times between 30^th^ April 2020 and 11^th^ July 2022. One panel assessed psychological distress (Kessler-10) and well-being (short Warwick Edinburgh Mental Well-being scale) *n* = 313, and one panel assessed resilience (10-item Connor-Davidson Resilience Scale^©^) *n* = 583. A linear panel regression model with random effects assessed the temporal patterns for psychological distress, well-being, and resilience.

**Results:**

Mean psychological distress scores (Kessler-10) were relatively stable over time and were not statistically significantly worse than baseline at any follow-up. Well-being scores improved over time and were significantly better than baseline by the third survey (22^nd^ Jul-18th Aug 2020) (0.54 *p* = 0.007, Cohen’s _*d*_ 0.12). Scores had worsened by the last survey (27^th^ June-11^th^ July 2022) but were not significantly different from baseline 0.20 *p* = 0.30. Resilience scores declined over time. Scores at both follow-ups (14th Aug- 4th Sep 2020 and 27^th^ June-11^th^ July 2022) were statistically significantly lower than baseline: -1.69 *p* < 0.001 (Cohen’s _*d*_ 0.25) and -0.96 *p* = 0.006 (Cohen’s _*d*_ 0.14), respectively.

**Conclusions:**

Our study joins a body of work measuring the longitudinal effects of COVID-19 on population mental health and resilience. While, the magnitude of the effect related to resilience decline is small, our findings indicate that particular attention should be given to ongoing population surveillance, with the aim of maintaining good health and well-being.

**Supplementary Information:**

The online version contains supplementary material available at 10.1186/s12889-023-17230-1.

## Background

In 2019, COVID-19 outbreaks led to the rapid adoption of global border closures, remote working, and other social distancing practices [1]. These containment measures worked to successfully flatten COVID-19 cases but have not been without repercussions. Population segregation strategies socially isolated individuals, a known risk factor for depression [2]; border restrictions disrupted manufacturing, international supply chains, and other industries, leading to an estimated 114 million job losses or reduced working hours [3] and school closures placed parents under pressure to support home schooling [4]. Overall, these factors have had a profound impact on the mental health and well-being of populations [2, 5, 6].

In times of adversity, population resilience or the capacity to recover from a disaster is crucial. The importance of building and sustaining resilience for population health and well-being is recognised by the World Health Organization [7]. Thus, in addition to population mental health it is another important metric that can provide crucial insights into how individuals and communities cope with adversity and recover over time. By assessing resilience, mental health and well-being, planners can allocate resources more effectively, tailor support services to different needs, and identify vulnerable groups requiring targeted interventions. Furthermore, measuring resilience can inform strategies for building stronger, more adaptive communities in the face of future disasters.

Historically, past pandemics and natural disasters are often followed by increased anxiety, depression, and post-traumatic stress disorders in the general population [8–11]. For example, the H1N1 Swine Flu outbreak in 2009 [12] and the Great East Japan Earthquake in 2011 [13] led to large-scale incidences of depression and other mental illnesses in the community. A similar rise in mental health disorders has also followed COVID-19 [14–18]. Population studies have identified several risk factors for mental health decline during the pandemic, including younger age, having children, female gender, unemployment, specific ethnicities, and financial instability [16, 19–24]. However, meta-analyses of longitudinal studies indicate a lack of data from certain regionsand very few longitudinal studies that continued beyond the first year of the pandemic [25–28].

We aimed to address the gaps in the literature by tracking changes in psychological distress, well-being, and resilience over time in a multi-ethnic Singaporean population. Specifically, we investigated how mental health and resilience evolved during the first year of the pandemic (2020) as local restrictions changed and what the population situation is two-years later (2022). In addition, we assessed what characteristics were associated with mental health and resilience scores.

## Methods

We used data from the “Strengthening our community’s resilience against threats from emerging infections” (SOCRATES) cohort [29]. The SOCRATES cohort was set up in 2019, prior to the emergence of COVID-19 to assess public knowledge and perceptions of infectious disease outbreaks. This study is reported according to the STrengthening the Reporting of OBservational studies in Epidemiology (STROBE) checklist [30].

### Local lock down measures

**Fig. 1:**
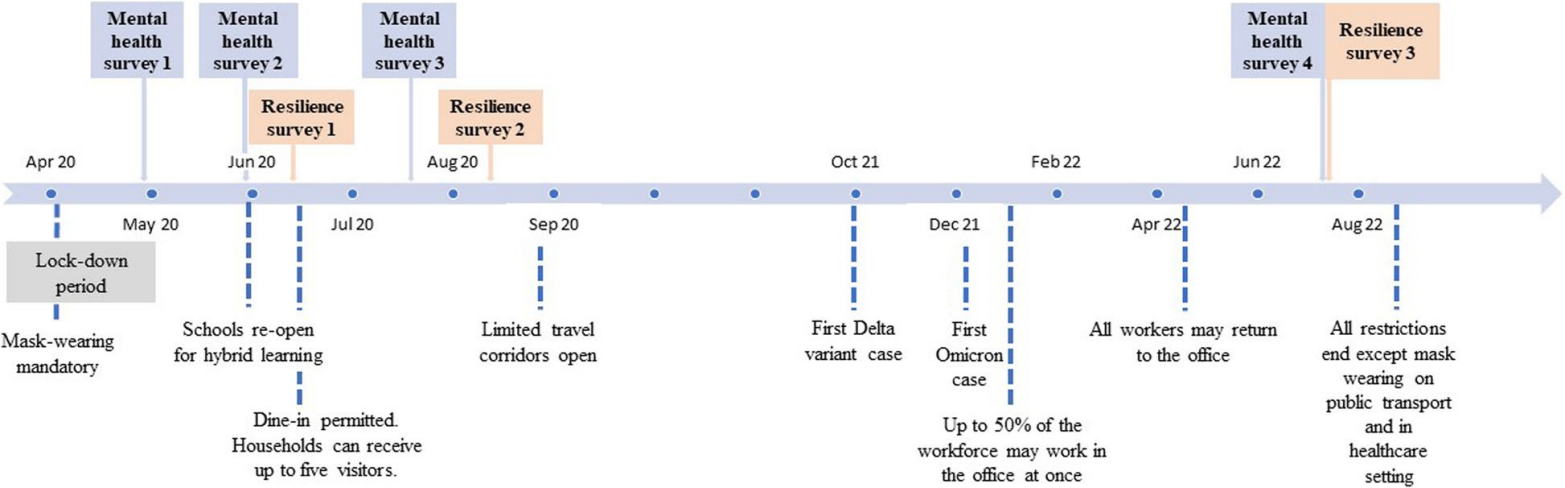
Summary of key pandemic events and corresponding survey times

In Singapore, a countrywide lock-down (locally known as ‘circuit breaker’) was first implemented in early April 2020 [31], during which time residents were required to remain indoors (except for essential trips), wear masks whenever outside the home, work from home or attend school remotely, and avoid interactions with other households. Many elders and adults living alone became isolated, families and children had to adapt to home-schooling, childcare arrangements were disrupted, and unemployment rose. Following the decline of COVID-19, a phased relaxation of containment measures continued until August 2022 (Fig. 1).

### Recruitment strategy

The SOCRATES cohort uses a probability sampling approach and is intended to be nationally representative of the Singaporean population. Residential estates (primary sampling units) were chosen across Singapore to include a diverse range of areas. Within each residential estate, households were randomly selected for the cohort. Up to four residents per household can join the cohort. Recruitment to the SOCRATES cohort occurs through a combination of door-to-door visits, word of mouth and social media posts. Face-to-face recruitment was replaced with an electronic format when COVID-19 reached Singapore in January 2020. The cohort also used a snowballing approach, in that participants could recommend others to join. The profile of the recruited participants is continually assessed to ensure balanced representation of the population. SOCRATES was launched on 27^th^ June 2019, and on 24^th^ January 2020 the first COVID-19 case was reported in Singapore. On 30^th^ April 2020, COVID-19-related questions were included in the survey, and in subsequent rounds following key events throughout the pandemic [29].

Only Singapore citizens and permanent residents are eligible to participate. Enrolled participants are registered, given a unique identifier, and then asked to complete a survey on their baseline characteristics. Through an app, participants are invited to complete surveys. Respondents receive a five Singapore dollar incentive for each survey they complete. Surveys are available in the main local languages (English, Mandarin, and Malay). All survey questions were mandatory.

### Survey timing

Questions relating to mental health (Kessler-10) and well-being (Short Warwick Edinburgh Mental Well-being Scale (SWEMWS)) were included four times between 30th April 2020 to 11th July 2022. To assess participants’ resilience the Connor-Davidson Resilience Scale-10 (CD-RISC) was included in the survey three times between 11^th^ June 2020 to 11^th^ July 2022 (Table 1). The last survey, conducted between 27^th^ June to 11^th^ July 2022, was used to assess population mental health, well-being and resilience two years after COVID-19 restrictions were implemented. The survey was timed to coincide with key events relating to COVID-19 (Fig. 1) [32].

**Table 1:** Survey time points and response rate between 30^th^ April 2020 to 11^th^ July 2022

Sample n and response rate (%)	Survey dates 2020					Survey 2022
	30^th^ Apr-14^th^ May 688 (86)	29^th^ May-11^th^ Jun 864 (78)	11^th^ Jun-2^nd^ Jul 1050 (87)	22^nd^ Jul- 18^th^ Aug 1534 (94)	14^th^ Aug- 4^th^ Sep 1634 (92)	27^th^ June-11^th^ July 2006 (69)
Kessler-10	X	X		X		X
SWEMWS	X	X		X		X
CD-RISC-10			X		X	X

*Abbreviations*: *SWEMWS* Short Warwick Edinburgh Mental Well-being Scale, *CD-RISC 10* Connor-Davidson Resilience Scale-10

### Outcome measures

Mental health was assessed using the Kessler-10, a self-reported instrument that measures emotional state over the prior four weeks [33]. The instrument consists of ten questions using a 5-point Likert scale and generates a global measure of distress. The distress score range between ten to fifty, with lower scores indicating lower psychological distress [34]. The SWEMWS was used to assess well-being and psychological functioning over the last two weeks [35]. The instrument consists of seven questions on a 5-point Likert scale, which generates a summary score between seven and thirty-five. Higher scores indicate greater mental well-being. All scores were converted to metric scores before analyses. The abbreviated 10-item CD-RISC was used to assess resilience in the population. The instrument includes ten questions on personality, stress, and coping, graded on a 5-point Likert scale. A summary score is generated between zero and forty, with higher scores indicating greater resilience [36]. We obtained officially validated English, Chinese and Malay versions of the Kessler-10, CR-RISC, and SWEMWS instruments for this study [37–39]. For SWEMWS, the creators do not hold an official Malay language version, however a published validation study conducted in Malaysia (a culturally close country to Singapore) does exist [40].

### Statistical analysis

Analyses were performed in STATA v15.0 (STATA Corp, College Station, Texas, USA). Summary statistics are presented as means with standard deviations (SD) or proportions. Mean and SD were calculated for each survey period and instrument.

We used a balanced panel approach. That is, only participants with data from the first mental health survey and data at each subsequent survey were included in the analyses *n* = 313. Resilience questions were launched later, following the end of the circuit breaker, and were analysed in a separate cohort (also a balanced panel). As recruitment was rolling and new participants joined each month, the resilience cohort was larger (*n* = 583). Demographics of the analysis cohort, full cohort, non-responders, and dropouts at each survey timing are included in Supplement 1. A dropout was defined as a participant who didn’t respond at a survey wave and all subsequent survey waves.

A linear panel regression model with random effects was used to assess the temporal relationships of: i) COVID-19 and mental health, well-being and resilience and; ii) to identify if participant characteristics (age, sex: male/female, ethnicity: Chinese, Indian, Malay, Others, education level: primary level or below, O-level/N-level, Diploma/A-level, higher degree, monthly household income level: < S$1,000, S$1,000–4,999, S$5,000–8,999, S$9,000–19,999, ≥ S$20,000, employment status: employed, in school, self-employed, not employed or in school, living alone or the presence of one or more medical conditions) were associated with mental health, well-being and resilience. We hypothesised that younger age, having children, female gender, unemployment, and minority groups would be at risk of poorer mental health and well-being scores [16, 19–24]. The baseline survey measure was used as the reference group in each panel regression. We calculated the Cohen’s _*d*_ for paired samples to interpret the magnitude of any clinical effects if statistically significant differences from baseline scores were observed [41]. An effect size of 0.2 is considered small, 0.5 moderate, and 0.8 large [41]. Statistical significance was set at *p* < 0.05.

### Patient and public involvement

No patients or members of the public were involved in this study.

## Results

A total of 313 participants completed all survey time points for the mental health measures, and 583 respondents completed the resilience assessment at all time points. Participant characteristics are reported in Table 2. Participant characteristics did not substantially differ between the cohorts, except for age which was significantly higher in the resilience cohort (*p* = 0.01). Respondents were mainly of Chinese ethnicity, well-educated, and employed in both cohorts.

**Table 2:** Participant characteristics

Participant Characteristics	Mental health measures *N* = 313	Resilience measure *N* = 583
Mean age, years (SD)	41.72 (14.59)	44.23 (14.31)
Male, n (%)	157 (50)	281 (48)
Ethnicity, n (%)		
Chinese	280 (90)	536 (91)
Malay	17 (5)	18 (3)
Indian	12 (4)	28 (5)
Other	4 (1)	7 (1)
Education, n (%)		
Primary of below	8 (2)	7 (1)
O-level/N-level	38 (12)	68 (12)
Diploma/A-level	96 (31)	154 (26)
Higher degree	171 (55)	360 (61)
Household income S$, n (%)		
< 1,000	19 (6)	43 (7)
1,000–4,999	83 (27)	141 (24)
5,000–8,999	92 (29)	165 (28)
9,000–19,999	99 (32)	196 (33)
≥ 20,000	20 (6)	44 (8)
Employment status, n (%)		
Employed	198 (63)	354 (60)
Schooling	26 (8)	43 (7)
Self-employed	33 (11)	66 (11)
Not employed nor schooling	56 (18)	126 (22)
Living alone, n (%)	16 (5)	40 (7)
≥ 1 medical condition, n (%)	65 (21)	145 (25)

*Abbreviations*: *SD* Standard Deviation

### Mental health, well-being and resilience overtime

Mean psychological distress scores (Kessler-10) were relatively stable over time (Table 3) and were not statistically significantly different from baseline (30^th^ Apr-14^th^ May 2020) at any follow-up point (29th May-11th Jun 2020, 22nd Jul- 18th Aug 2020, 27^th^ June-11^th^ July 2022.

**Table 3:** Kessler-10, Short Warwick Edinburgh Mental Well-being Scale and Connor Davidson Resilience Scale-10 scores at each survey time point, mean (SD)

	Survey dates 2020			Survey dates 2022		
	Baseline 30^th^ Apr-14^th^ May	29^th^ May-11^th^ Jun	11^th^ Jun-2^nd^ Jul	Baseline 22^nd^ Jul- 18^th^ Aug	14^th^ Aug- 4^th^ Sep	27^th^ June-11^th^ July
Mean Kessler-10 score (SD)	19.07 (7.17)	18.69 (7.51)	18.64 (7.72)	-	-	19.01 (8.04)
Mean SWEMWS score (SD)	22.49 (4.11)	22.62 (4.17)	23.03 (4.54)	-	-	22.69 (4.62)
Mean CD-RISC 10 score (SD)	-	-	-	26.39 (6.47)	24.70 (6.82)	25.43 (7.05)

*Abbreviations*: *SWEMWS* Short Warwick Edinburgh Mental Well-being Scale, *CD-RISC 10* Connor-Davidson Resilience Scale-10, *SD* Standard Deviation

Well-being scores improved over time (Table 3) and were significantly better than baseline by the third survey (22^nd^ Jul-18th Aug 2020) (0.54 *p* = 0.007, Cohen’s _*d*_ 0.12). Scores had worsened by the last survey (27^th^ June-11^th^ July 2022) but were not significantly different from baseline 0.20 *p* = 0.30.

Resilience scores declined over time (Table 3). Scores from both follow-up surveys (14th Aug- 4th Sep 2020 and 27^th^ June-11^th^ July 2022) were statistically significantly lower than baseline: -1.69 *p* < 0.001 (Cohen’s _*d*_ 0.25) and -0.96 *p* = 0.006 (Cohen’s _*d*_ 0.14), respectively.

### Characteristics associated with mental health, well-being and resilience scores

Factors positively associated with better psychological distress scores were increasing age -0.14 *p* < 0.001 and Malay ethnicity -5.23 *p* = 0.01. Factors positively associated with better well-being scores were increasing age 0.06 *p* = 0.001 and Malay ethnicity 2.35 *p* = 0.01. Factors positively associated with better resilience scores were increasing age (0.07 *p* < 0.001), Malay ethnicity (3.08 *p* = 0.006) and being of the other ethnicity group 3.52 *p* = 0.04. No other variables were statistically significantly related to psychological distress, well-being or resilience scores.

## Discussion

Since the first documented case of COVID-19, evidence syntheses have shown small but noticeable impacts on the mental health of general populations. However, very few longitudinal studies have continued to evaluate the impact of COVID-19 beyond the first year of the pandemic [14–18]. Building on existing studies, we evaluated mental health, well-being, and resilience during the first year of the pandemic (2020) and two-years later (2022) [14–18]. We found that despite the resolution of COVID-19 containment measures, mean Kessler scores remained static and well-being scores improved during 2020 but declined in 2022. However, without pre-pandemic baseline values for Kessler and SWEMWS it is difficult to assess whether this is a time-trend or the effect of COVID-19. Conversely, resilience scores decreased continually over time and were lower than pre-pandemic levels [42], indicating a reduced capacity to recover from adversity in the local population despite local support initiatives. While, the magnitude of the effect is small, our study highlights the need for ongoing population surveillance.

The ability of societies to cope with and recover from traumatic events, also termed resilience, is linked to individual traits and the environment within which individuals exist. Yip et al*.* (2021) describes community resilience in terms of five domains: Physical and psychological health, communication, social connectedness, integration or involvement of organisations, and social responsibility [43]. Identifying environmental stressors (e.g., financial insecurity) and at-risk groups can help policymakers implement targeted interventions. For example, the Singaporean grants scheme supported low-income families against loss of earnings [44]. However, we still observed resilience scores lower than pre-pandemic levels (mean score 26.5) in the population [42]. Possible explanations could be uncertainty as to how the disease will evolve, whether restrictions will be re-introduced, and economic instabilities. However, further work is needed to understand the exact mechanisms behind resilience decline.

Compared to other regional countries [45–49] and elsewhere [26, 27, 50–53], female sex was not predictive of poorer mental health in our study. It has been well reported that women were disproportionality impacted by job loss or loss of hours during the pandemic [54]. Women were also more likely than men to take on household responsibilities like childcare and home schooling [50]. Economic constraints and the burden of greater household responsibilities have been associated with poorer mental health in women during the pandemic [50]. It is possible a similar effect was not observed in Singapore due to the low-income grant schemes [44], which alleviated financial strain [55], cultural norms (i.e., close family structure and the availability of childcare among other family members), and the availability of domestic helpers locally, who can assist with household responsibilities.

Another factor associated with mental health decline reported elsewhere but not in our study, was being of an ethnic minority group. Studies from the United Kingdom and the United States identified poorer mental health in ethnic minorities during the pandemic [23, 56, 57]. We found no such association in our study or in another local study of mental health in low-skilled dormitory-based migrant workers in Singapore [58]. Financial security may offer one explanation. Ethnic minorities in other studies typically work in unskilled, lower-income roles (e.g., service sector), which were disproportionately impacted by job losses or reduced hours when COVID-19 hit. Locally, these same financial pressures may have been alleviated by introducing initiatives to protect jobs, grants to support low-income households and government support to help companies remain solvent [43]. Our study also observed a higher psychological resilience in Malays (one ethnic minority in Singapore), a phenomenon also seen before the pandemic [42]. Higher psychological resilience can protect individuals from mental health decline. The potential reasons for this are beyond the scope of this study but warrant further investigation.

Decisive measures to control the spread of COVID-19 were instrumental during the initial outbreak of COVID-19, but the unintended consequences of these measures cannot be ignored. By implementing population-wide screening initiatives it is possible to detect emerging trends and intervene early, allocate resources if and where needed, and inform future mental health care policies. Accordingly, the World Health Organization (WHO) has called for mental health reforms, following an estimated 25% increase in the global prevalence of anxiety and depression observed in 2020 [59]. Recommendations include increased funding to expand capacity, upskilling of community providers to aid in screening and treatment, a refocus on preventative care and protection of vulnerable groups and the leveraging of technology to meet these goals and expand access [59]. Recent developments in mental health care including the rapid expansion of virtual services and the use of big data analytics to identify those at risk, may help to deliver some of the desired WHO reforms, but their effectiveness and safety still need to be established [60–62].

### Limitations

While the SOCRATES survey aims to assess public knowledge and perceptions of infectious diseases, the responses may not represent the wider Singaporean population. The limitation of an electronic survey format may also have resulted in a biased sample agreeing to participate. For instance, those that are more technology literate. Mental health and well-being assessments are also not routinely reported locally. Thus, we cannot compare our findings to the pre-pandemic state, except for resilience for which we had pre-pandemic data. Furthermore, we cannot rule out memory effects (i.e., a response influenced by prior responses to the same question). However, these may be minimised for the Kessler, SWEMWS and CD-RISC-10 instruments, which ask questions anchored to a specific period, reducing the influence of past responses. Finally, all survey questions were mandatory to minimise missing data, however, we acknowledge this may have introduced response bias into the study.

## Conclusions

We observed a small determinantal impact on the resilience of the general population in our study. Policymakers should strive to identify and alleviate contextual factors that continue to create stress to prevent resilience decline. These may include social, economic, and health-related factors. Furthermore, investment in population surveillance is warranted to aid in decision-making when allocating finite resources for population mental health and well-being.

### Supplementary Information

**Additional file 1: Table 1.** Demographics of analysis cohort, full cohort, and non-responders at each survey timing (Kessler-10 and Short Warwick Edinburgh Mental Well-being Scale questions)*. ***Table 2*.*** Demographics of analysis cohort, full cohort, and non-responders at each survey timing (Connor-Davidson Resilience Scale-10 questions). **Table 3.** Demographics of unique dropouts at each study wave.

## Data Availability

Deidentified data are available upon reasonable request. The corresponding author, Jennifer Sumner, can be conducted for any data-related requests.

## Abbreviations

SD: Standard Deviation
SOCRATES: Strengthening our community’s resilience against threats from emerging infections”
STROBE: STrengthening the Reporting of OBservational studies in Epidemiology
WHO: World Health Organization
