# Supplementary material for: Mental health and resilience after the covid-19 pandemic: a multi-ethnic longitudinal survey
Source: BMC Public Health. 2023 Nov 25;23:2340. doi: 10.1186/s12889-023-17230-1 (PMC10676608; doi:10.1186/s12889-023-17230-1)
Supplement: Supplementary file 1 — Additional file 1: Table 1. Demographics of analysis cohort, full cohort, and non-responders at each survey timing (Kessler-10 and Short Warwick Edinburgh Mental Well-being Scale questions). Table 2. Demographics of analysis cohort, full cohort, and non-responders at each survey timing (Connor-Davidson Resilience Scale-10 questions). Table 3. Demographics of unique dropouts at each study wave. [file 12889_2023_17230_MOESM1_ESM.docx]

**Supplement 1: Profile of survey non-responders**

**Table 1 Demographics of analysis cohort, full cohort, and non-responders at each survey timing (Kessler-10 and Short Warwick Edinburgh Mental Well-being Scale questions)**

| **Characteristics** | **Analysis cohort n=313**  30^th^ Apr- 14^th^ May | **Full cohort n=591**  30^th^ Apr- 14^th^ May | **n=71 non-responders on**  29^th^ May- 11^th^ Jun | **n=59 non-responders on** 22^nd^ Jul-  18^th^ Aug | **n=256 non-responders on**  27^th^ June-11^th^ July |
| --- | --- | --- | --- | --- | --- |
| Mean age, years (SD) | 41.72 (14.59) | 39.48 (14.82) | 36.15 (12.86) | 33.42 (13.13) | 37.16 (15.00) |
| Male, n (%) | 157 (50) | 225 (38) | 34 (48) | 28 (47) | 112 (44) |
| Ethnicity, n (%)  Chinese  Malay  Indian  Other | 280 (90)  17 (5)  12 (4)  4 (1) | 493 (83)  39 (7)  45 (8)  14 (2) | 46 (65)  11 (16)  13 (18)  1 (1) | 40 (68)  8 (13)  10 (17)  1 (2) | 197 (77)  20 (8)  29 (11)  10 (4) |
| Education, n (%)  Primary or below  O-level/N-level  Diploma/A-level  Higher degree | 8 (2)  38 (12)  96 (31)  171 (55) | 2 (1)  93 (15)  195 (33)  301 (51) | 2 (3)  13 (18)  18 (25)  38 (54) | 2 (3)  10 (17)  26 (44)  21 (36) | 2 (1)  47 (18)  92 (36)  115 (45) |
| Household income S$, n (%)  <1,000  1,000-4,999  5,000-8,999  9,000-19,999  ≥20,000 | 19 (6)  83 (27)  92 (29)  99 (32)  20 (6) | 35 (6)  160 (27)  174 (29)  194 (33)  28 (5) | 2 (3)  19 (27)  21 (29)  26 (37)  3 (4) | 2 (3)  20 (34)  17 (29)  19 (32)  1 (2) | 15 (6)  75 (29)  72 (28)  87 (33)  7 (3) |
| Employment status, n (%)  Employed  Schooling  Self-employed  Not employed nor schooling | 198 (63)  26 (8)  33 (11)  56 (18) | 354 (60)  83 (14)  66 (11)  88 (15) | 50 (70)  6 (9)  8 (11)  7 (10) | 35 (59)  12 (21)  9 (15)  3 (5) | 137 (54)  54 (21)  33 (13)  32 (12) |
| Living alone, n (%) | 16 (5) | 25 (4) | 3 (4) | 1 (2) | 7 (3) |
| ≥1 medical condition, n (%) | 65 (21) | 118 (20) | 14 (20) | 19 (32) | 50 (20) |

**Table 2 Demographics of analysis cohort, full cohort, and non-responders at each survey timing (Connor-Davidson Resilience Scale-10 questions)**

| **Characteristics** | **Analysis cohort n=583**  11^th^ Jun- 2^nd^ Jul | **Full cohort  n=916**  11^th^ Jun- 2^nd^ Jul | **n=61 non-responders on**  14^th^ Aug-  4^th^ Sep | **n=312 non-responders on**  27^th^ June- 11^th^ July |
| --- | --- | --- | --- | --- |
| Mean age, years (SD) | 44.23 (14.31) | 42.37 (15.03) | 37.63 (13.77) | 38.94 (15.74) |
| Male, n (%) | 281 (48) | 356 (39) | 23 (38) | 128 (41) |
| Ethnicity, n (%)  Chinese  Malay  Indian  Other | 536 (91)  18 (3)  28 (5)  7 (1) | 807 (88)  38 (4)  50 (6)  21 (2) | 42 (69)  11 (18)  6 (10)  2 (3) | 258 (83)  19 (6)  21 (7)  14 (4) |
| Education, n (%)  Primary or below  O-level/N-level  Diploma/A-level  Higher degree | 7 (1)  68 (12)  154 (26)  360 (61) | 3 (11)  120 (13)  285 (31)  508 (55) | 2 (3)  5 (8)  28 (46)  26 (43) | 2 (1)  52 (16)  115 (37)  143 (46) |
| Household income S$, n (%)  <1,000  1,000-4,999  5,000-8,999  9,000-19,999  ≥20,000 | 43 (7)  141 (24)  165 (28)  196 (33)  44 (8) | 59 (6)  229 (25)  260 (28)  306 (34)  62 (7) | 0  18 (29)  15 (25)  24 (39)  4 (7) | 16 (5)  84 (27)  91 (29)  105 (34)  16 (5) |
| Employment status, n (%)  Employed  Schooling  Self-employed  Not employed nor schooling | 354 (60)  43 (7)  66 (11)  126 (22) | 521 (57)  110 (12)  112 (12)  173 (19) | 35 (58)  9 (15)  10 (16)  7 (11) | 161 (52)  65 (21)  42 (13)  44 (14) |
| Living alone, n (%) | 40 (7) | 49 (5) | 0 | 9 (3) |
| ≥1 medical condition, n (%) | 145 (25) | 203 (22) | 17 (28) | 64 (21) |

**Table 3 Demographics of unique dropouts at each study wave**

| **Characteristics** | **30^th^ Apr- 14^th^ May 2020**  **n=1** | **29^th^ May- 11^th^ Jun 2020**  **n=0** | **11^th^ Jun- 2^nd^ Jul  2020**  **n=4** | **22^nd^ Jul-  18^th^ Aug 2020**  **n=17** | **14^th^ Aug-  4^th^ Sep**  **2020**  **n=10** | **27^th^ June- 11^th^ July 2022**  **n=269** |
| --- | --- | --- | --- | --- | --- | --- |
| Mean age, years (SD) | 64 |  | 27.5 (6.19) | 35.05 (11.45) | 38.7 (17.01) | 37.07 (15.11) |
| Male, n (%) | 0 (0) |  | 2 (50) | 12 (70) | 4 (40) | 113 (42) |
| Ethnicity, n (%)  Chinese  Malay  Indian  Other | 1 (100)  0 (0)  0 (0)  0 (0) |  | 3 (75)  1 (25)  0 (0)  0 (0) | 15 (88)  0 (0)  2 (12)  0 (0) | 6 (60)  2 (20)  3 (20)  0 (0) | 217 (81)  17 (6)  25 (9)  10 (4) |
| Education, n (%)  Primary or below  O-level/N-level  Diploma/A-level  Higher degree | 0 (0)  1 (100)  0 (0)  0 (0) |  | 1 (25)  1 (25)  1 (25)  1 (25) | 0 (0)  0 (0)  9 (53)  8 (47) | 0 (0)  2 (20)  4 (40)  4 (40) | 10 (4)  23 (9)  98 (36)  138 (51) |
| Household income S$, n (%)  <1,000  1,000-4,999  5,000-8,999  9,000-19,999  ≥20,000 | 0 (0)  0 (0)  0 (0)  1 (100)  0 (0) |  | 0 (0)  3 (75)  1 (25)  0 (0)  0 (0) | 0 (0)  5 (28)  6 (36)  6 (36)  0 (0) | 0 (0)  4 (40)  3 (30)  2 (20)  1 (10) | 15 (6)  54 (20)  79 (29)  102 (38)  19 (7) |
| Employment status, n (%)  Employed  Schooling  Self-employed  Not employed nor schooling | 0 (0)  0 (0)  0 (0)  1 (100) |  | 1 (25)  0 (0)  1 (25)  2 (50) | 9 (53)  3 (18)  4 (23)  1 (6) | 7 (70)  2 (20)  0 (0)  1 (10) | 140 (52)  59 (22)  33 (12)  37 (14) |
| Living alone, n (%) | 0 (0) |  | 0 (0) | 0 (0) | 0 (0) | 10 (4) |
| ≥1 medical condition, n (%) | 0 (0) |  | 1 (25) | 5 (29) | 5 (50) | 54 (20) |
| *Dropouts were defined as those who didn’t respond at a survey wave or any subsequent survey waves* | | | | | | |
